# Combined unicompartmental knee arthroplasty and anterior cruciate ligament reconstruction in knees with osteoarthritis and deficient anterior cruciate ligament

**DOI:** 10.1186/s12891-016-1186-5

**Published:** 2016-08-05

**Authors:** Shaoqi Tian, Bin Wang, Yuanhe Wang, Chengzhi Ha, Lun Liu, Kang Sun

**Affiliations:** 1 Department of Orthopaedics, the Affiliated Hospital of Qingdao University, No. 1677 Wutaishan Road, Huangdao District, Qingdao, Shandong 266000 China; 2 Department of Orthopaedics, Qingdao 3rd People’s Hospital, Qingdao, 266000 China

**Keywords:** Osteoarthritis, Knee, Anterior cruciate ligament reconstruction, Unicompartmental knee arthroplasty, Oxford III prosthesis, Clinical outcomes

## Abstract

**Background:**

Relative young and more active patients with osteoarthritis (OA) of the isolated medial femorotibial compartment in conjunction with anterior cruciate ligament (ACL) deficiency are difficult to treat. The aim of this study was to explore the early clinical outcomes of combined Oxford unicompartmental knee arthroplasty (UKA) and ACL reconstruction for the patients presenting ACL deficiency and isolated OA of the medial compartment.

**Methods:**

Twenty-eight patients were included into the study. All patients were treated by combined Oxford UKA and ACL reconstruction. Plain radiographs in the antero-posterior and lateral view and long-leg standing radiographs were routinely performed prior to and after surgery. Stress radiographs in valgus were additionally available in order to verify the well-preserved lateral compartment. The varus deformity of the knee prior to surgery and the valgus degree after surgery, the posterior slope of the tibial component and the range of motion (ROM) of the knee after surgery were measured and recorded. Clinical evaluations include Oxford Knee Score (OKS), Knee Society Score (KSS-clinical score; KSS-function score) and Tegner activity score.

**Results:**

All the patients were followed up for 52 ± 8 months. The leg alignment showed 3.1 ± 0.6° of varus deformity prior to surgery and 4.0 ± 0.7° of valgus after surgery. The OKS, KSS and Tegner activity score improved significantly after surgery (*P* < 0.05). The mean ROM of the operated knee was 123.5 ± 2.8° at the last follow-up. The posterior slope of the tibial component was 3.9 ± 1.2°. A significant correlation was found between them according to the Pearson’s correlation (*r* = 0.39, *P* = 0.03). There were 2 patients (7 %) with the complication of mobile bearing dislocation, and a second operation of replacing a thicker mobile bearing was performed for them.

**Conclusion:**

The early clinical data have shown that combined surgery of UKA and ACL reconstruction has revealed promising results. However, long-term follow-up studies should be done in these patients.

**Trial registration:**

Current trial ISRCTN24663935 (Retrospectively registered on 21 July 2016).

## Background

Unicompartmental knee arthroplasty (UKA) is commonly used for the treatment of isolated, compartmental osteoarthritis (OA) of the knee. It has shown to be a good and less invasive alternative to total knee arthroplasty (TKA) [3]. Clinical outcomes have shown many advantages of UKA [13, 20, 23, 24]. The procedure has relatively strict indications and contraindications because of higher failure rates in the presence of several clinical variables. Anterior cruciate ligament (ACL) deficiency is a contraindication for the procedure. As it may cause abnormal knee kinematics and is associated with a tenfold increase in surgical failures, such as aseptic loosening of the tibial component [5, 12]. Kozinn and Scott believed ACL deficiency should be a relative contraindication to fixed bearing UKA [12]. Goodfellow and O’Connor reported higher failure rates with mobile bearing implants in knees with ACL deficiency [5].

Relative young and more active patients with OA of the isolated medial femorotibial compartment in conjunction with ACL deficiency are difficult to treat. Classic treatment options have included ACL reconstruction, high titial osteotomy (HTO), combined ACL reconstruction and HTO, and TKA. However, patients with an intact lateral and patello-femoral compartment may not require TKA. Due to the high complication rate seen HTO or HTO with ACL reconstruction [16], patients may also be reluctant to accept the options. Very few papers reported the treatment option of combined UKA and ACL reconstruction [13, 21, 30, 31]. Maybe as performing UKA with ACL reconstruction is technically demanding [15, 26, 28]. They could be interfered with each other.

The aim of the study was to follow a consecutive group of patients who received a combined procedure of UKA and ACL reconstruction. We hypothesized that the combined procedure would get outcome compared with isolated UKA according to the literature.

## Methods

From January 2008 to January 2014, a total of 28 patients with isolated medial femorotibial compartment OA secondary to ACL deficiency and primary isolated medial femorotibial OA with acute ACL injury were prospectively evaluated. Patients were offered Oxford UKA and ACL reconstruction if they had significant symptoms and presented with isolated end-stage disease. The hospital’s institutional review board approved the project and the written informed consent was obtained from all the patients. The inclusion criteria were according to the indications of the UKA except the intact ACL [6]. The patients were diagnosed of having isolated medial femorotibial compartment OA and ACL deficiency according to medical history, symptoms, physical and radiographic examinations. The ACL deficiency needed to be further confirmed during the operation. The primary complaint of all the patients included was instability of the knee and pain located in the medial femorotibial compartment. All the patients should be relatively younger and have higher activity level. Among the 28 patients (28 knees), there were 25 patients with primary ACL deficiency and secondary medial femorotibial OA, 3 patients with isolated medial femorotibial OA and acute ACL injury, the ACL injury happened within 6 weeks during sports. All these patients have no previous surgery on their knees. There were 15 left knees and 13 right knees, and 18 women and 10 men, with a mean age of 50.5 ± 3.5 years old (41 to 61) at the time of operation. The patients’ pre-operative Oxford Knee Score (OKS), Knee Society Score (KSS) and Tegner activity score were shown in Table 1.

**Table 1:** Comparing of OKS, KSS and Tegner scores pre-operation and at the last FU for the operated knees (*n* = 28, M ± SD)

Time point	OKS	KSS clinical score	KSS function score	Tegner activity score
Pre-operation	31 ± 7.1	60.4 ± 7.1	63.7 ± 6.5	4.4 ± 1.2
Last FU	43 ± 4.2	84.5 ± 6.3	86.9 ± 5.3	5.3 ± 0.8

*P* < 0.05

### Radiological examination and assessment

The pre-operative anteroposterior radiographs in 15° flexion, lateral, femoral-patellar in 30° flexion view and long-leg standing radiographs were routinely performed. Stress radiographs in valgus were additionally available in order to verify the well-preserved lateral compartment. The varus deformity of the knee prior to surgery, the valgus degree after surgery and the posterior slope of the tibial component were measured and recorded. The posterior slope of the tibial component was defined as an angle between the posterior inclination of the tibial implant and a line perpendicular to the posterior tibial cortex as described in the literature [11]. MRI was not performed on a regular basis. Standardized post-operative radiographs were taken for assessing for evidence of component loosening, the presence, type and extent of radiolucencies, progression of OA in the lateral compartment, and tibial and femoral tunnel position. Radiolucencies were analyzed and classified as pathological or physiological based on descriptions given by Goodfellow et al. [8, 9]. Subsidence was considered to be evidence of loosening. Radiographs were reviewed independently by two observers who were blinded to the clinical outcomes. The inter-observer reliability for radiological assessment was also examined.

### Surgical technique

All procedures were carried out using a minimally invasive technique for the Oxford III UKA (Biomet China, Bridgend, United Kingdom) [25]. We adopted the hamstring tendon autograft to reconstruct the deficient ACL with the arthroscopy assisted. The ACL surgery was anatomic single-bundle reconstruction [4]. All the combined surgeries were done by the same experienced surgeon.

Patient lied in a supine position having a tourniquet on his thigh as proximal as possible. Diagnostic arthroscopy was performed, and the indication of the combined procedure finally confirmed. For the patients with the acute ACL injury, the femoral side remnants of the torn ACL were removed and the medial meniscus was resected, while reserving the tibial side ACL remnants. If there was lateral meniscus injury, partial meniscectomy or meniscoplasty was done meanwhile. Patella denervation was performed laterally during arthroscopy using a radiofrequency probe and medially during the subsequent open procedure. Both semitendinosus and gracilis tendons were used for ACL reconstruction. The tendons were harvested after a vertical skin incision over the pes anserinus. A double-loop semitendinosus and gracilis tendon graft was prepared. A medial parapatella approach was used, beginning at 1 cm medial to the medial pole of the patella and extending distally to the tibial tubercle. A medial capsulotomy was performed leaving the quadriceps tendon intact in order to get sufficient access to the medial femorotibial compartment. Osteophytes were removed from the patella, the medial condyle and the intercondylar notch. The location of the tibial canal was determined first, and then the bone was prepared for the tibial component of the UKA according to the technical manual [7]. The horizontal cut was followed by the vertical cut. Then a cannulated drill was used for the tibial canal in a size matching with the diameter of the ACL graft. An offset guide was used to identify the correct site for the femoral canal at the posterolateral notch, which also referred to the bony ridges or the ACL remnants. Finally, the femoral condyle was prepared for the implant. At that point, the trial components were inserted to check the flexion and extension gap. Then after pulling the ACL graft into the femur via the tibial canal and fixing the graft on the femoral site, the tibial base plate and the femoral component were cemented by stages, after that the bearing with the suitable size was installed. Repeated extension and flexion was performed to allow setting of the graft. At last the ACL graft was fixed on the tibial site. The ACL graft was fixed with the Endobutton (Smith&Nephew, USA) on the femoral site. The ACL graft was fixed on the tibial site by Intrafix tibial fastener system (DePuy Mitek). One drain was used at the end of surgery.

### Postoperative managements

The drainage tube was placed for no more than 24 h. Antibiotic was applied for patients twice in the first day after operation. Analgesic and anticoagulant drugs were used routinely. In the 2nd day after operation X-rays of the operated knee were taken according to the requirement. The patients performed exercises of quadriceps with initiative and straight leg rising since 6 h after operations. Meanwhile, the patients started to walk with assistance of walker or crutch to help part of weight loading. Full weight bearing began at the 2nd week.

### Follow-up and clinical evaluations

Follow-up (FU) was done at 1, 3, 6, 12 months after operations and each 1 year thereafter. The post-operative range of motion (ROM), KT-2000 arthrometer testing, the varus or valgus degree of the operated knee and the posterior slope of the tibial component were recorded. Clinical evaluations included Oxford Knee Score, the KSS-clinical score and KSS-function score and the Tegner activity score. The evaluations were done by two fellowship-trained orthopedic surgeons who were also blinded to the study.

### Statistical analysis

All data were presented as mean and standard deviation of the mean. Statistical analyses were performed using SPSS for Windows (version 18.0; SPSS Inc., Chicago, Illinois, USA). Pearson’s correlation was used to analyze the impact of the posterior slope of the tibial component on the range of motion. As the data of OKS, KSS and Tegner scores prior to and after surgery were found not to be normally distributed, Wilcoxon’s signed ranks test was used for the data. The significant level was set at *P* < 0.05.

## Results

All the patients completed the whole FU. The mean FU time was (52 ± 8) months (24–96 months). No patient felt instability of the knee after surgery any more. All patients had a side-to-side difference of less than 3 mm according to KT-2000. The mean length of the incisions was (5.1 ± 0.3) cm. No patient had the complication of fracture during operation, and there was no patient with the complications such as collapse of tibial plateau, infection, thrombosis, and aseptic loose. There were 2 patients (7 %) with the complication of mobile bearing dislocation, both of the patients were the cases included in the study in the early stage, and received the second operation of replacing a thicker mobile bearing. Then they satisfied with the outcome. The leg alignment showed 3.1 ± 0.6° of varus deformity prior to surgery and 4.0 ± 0.7° of valgus after surgery. The OKS, KSS and Tegner activity score improved significantly after surgery (*P* < 0.05) (Table 1).

The posterior slope of the tibial component was 3.9 ± 1.2°. The mean ROM of the operated knee in sagittal plane at the last FU was 123.5 ± 2.8°. A significant correlation was found between the posterior slope of the tibial component and the ROM (*r* = 0.39, *P* = 0.03), that means higher tibial slopes are correlated with more range of motion. All the interobserver reliabilities for the radiographic assessments were above 0.95.

At the last FU, there was no patient had evidence of component subsidence or pathological radiolucency. There were 10 components on the tibial site showing physiological radiolucency of a maximum of 1 mm.

## Discussion

### Inclusions and treatment options for the patients

In order to choose the right options for the patients with medial femorotibial OA and ACL deficiency, strict inclusion criteria should be made and the primary pathology should be also explored. As the consequences of ACL deficiency in association with medial femorotibial OA depends on the primary pathology. If arthritis is the primary problem, it tends to begin anteriorly in the medial compartment and then extend posteriorly and progressively damage the ACL [10, 18, 31]. By the time the ACL is destroyed the medial collateral ligament has shortened and the lateral side is usually damaged [2, 21]. For these patients the UKA combined with ACL reconstruction would be inappropriate, and the best option is a TKA. In contrast, if the ACL deficiency is the primary problem, the arthritic change tends to begin posteriorly in the medial compartment [14]. This is possibly due to recurrent episodes of giving way, in which posterior femoral subluxation in the medial compartment places a heavy load on the posterior part of the medial meniscus and underlying articular cartilage of the tibia, producing medial compartment OA. These patients tend to be relatively young and active, and the medial collateral ligament and lateral compartment are relatively normal. In such situations, the surgical treatment options are as follows: HTO with or without ACL reconstruction, UKA with or without ACL reconstruction, TKA or ACL reconstruction alone [16]. These treatment options also apply to the patients with primary isolated medial femorotibial OA combined with acute ACL injury.

Due to younger age and higher activity levels seen in these patients, bone conserving options are preferred with TKA not being recommended as the primary treatment option [27, 31]. Such patients can consider the options of HTO/UKA combined with ACL reconstruction. For the patients who report instability as their primary complaint, ACL reconstruction alone can be used as a reasonable, low co-morbility treatment option to improve symptoms prior to subsequent HTO or UKA, which is a definitive treatment option [32]. Both HTO and UKA have been demonstrated to be valid treatment options for the treatment of isolated medial OA, while the philosophy behind each technique is markedly different, the indications and outcomes of each technique are also different, and there is still lack of consensus in the literature as to which technique provides optimum outcomes for specific patient subgroups. But more and more papers reported that the rate of complications and revisions following HTO was higher than the rate seen in patients receiving UKA. So in this study we choose the UKA rather than HTO combined with ACL reconstruction as the treatments options for the patients.

### Clinical outcomes

From the study, satisfactory early and midterm clinical outcomes were obtained which was completely comparable to the data reported by other researchers. Krishnan SR, et al. reported the results of their studies, in which 9 patients were treated by the combined operation of UKA and ACL reconstruction, and were followed up for 2 years [13]. No revision was required in these patients and the mean KSS was as high as 196 points. Similar excellent clinical outcomes were reported by Pandit H, et al. In their study, 15 patients received the combined operation and the mean KSS was 195 points after a FU time of 2.5 years [21]. To be differently, both of the studies just reported the early clinical outcomes, while in our study the patients were followed up relatively longer time and the satisfactory results were reported with the KSS clinical and function score of 84.5 and 86.9 respectively. In 2012, Tinius M, et al. reported their midterm clinical data with a mean FU time of 50 months [30], which revealed promising results with the KSS clinical and function score of 84.1 and 83.4 respectively. Comparing with these results, our outcomes were much better. The definite reasons were not clear. It somewhat related to the different prosthesis the two studies adopted. In Tiniu M’s study all patients received fixed-bearing tibial component, while in the current study the mobile bearing Oxford phase III prosthesis was adopted. The mobile bearing could result in low wear and loosening rates according to the literature [17]. Weston-Simons JS et al. also reported their outcome of combined Oxford UKA and combined or sequential ACL reconstruction in 2012. Fifty-two patients were enrolled the study with a mean FU of 5 years. The OKS and Tegner activity score were improved from 28 and 2.5 to 41 and 3.5 respectively [31]. In our study, the OKS and Tegner activity score were improved from 31 and 4.4 to 43 and 5.3 respectively. In our study, the patients were more active and all the combined Oxford UKA and ACL reconstruction were done by the same experienced surgeon, and the hamstring tendons autograft were chosen for the patients. While in Weston-Simons JS’s study, patients received simultaneous or staged UKA and ACL reconstruction, and the operations were done by different surgeons. The grafts chosen for ACL reconstruction were also different, including hamstring and bone-patellar tendon-bone graft. Another study about the in vivo kinematics of the combined Oxford UKA and ACL reconstruction showed that the sagittal plane kinematics were nearly normal after combined UKA and ACL reconstruction [22]. This may also further explain why these knees in our study have good function and do not have tibial loosening in the FU time.

### Technique notes

One should keep in mind that these combined procedures are technically very demanding. Surgeons should have rich experience of doing procedures of UKA and ACL reconstruction. We know that the long-term success rate in UKA correlates well with the number of operations performed per year [7]. The same is true in ACL surgery [15]. By reviewing literature, the papers reporting about the procedures of UKA and ACL reconstruction for the patients were very few. So, here we made some technical key points which should keep in mind. The first is that the tibial canal should be positioned as lateral as possible in order to avoid weakening of the medial tibial plateau. The collapse of the medial tibial plateau was reported the most common mode of failure [1]. Secondly, after determining the location of the tibial canal and drilling the guide wire, the tibial canal was drilled after bone preparation for the tibial component being completed, in case the tibial cuts came too close to the tibial guide wire, there was still a chance for correcting the position of the guide wire. Another point is that for the ACL reconstruction we adopted anatomic single-bundle reconstruction technique, and the location of tibial and femoral canal sites referred to the remnant of ACL and the bone marks. The last is that after drilling the ACL tibial and femoral canal, preparing the bone bed for the UKA components, and checking the flexion and extension gap, next procedures are important. You should firstly pull the ACL graft into the femur via the tibial canal, followed by fixing the graft on the femoral site, and then installing the components, at last the ACL graft was fixed on the tibial site. In this sequence, the size of the mobile bearing could be chosen accurate and the suitable tension of the ACL graft could be guaranteed.

### Radiological assessment

In this study, the posterior slope of the tibial component was 3.9° and within the range as reported by others [11, 19]. Hernigou P, et al. recommended that the posterior slope of tibial component not exceed 7° [11]. They also reported the significant correlation between the anterior translation and the posterior slope of the tibial component. In our study, a significant correlation was found between the posterior slope and the ROM, which means that higher tibial slopes are correlated with more range of motion. However, one should keep in mind that the posterior slope of the tibial component should refer to the posterior slope pre-operation or that of the opposite knee, try to avoid increasing the posterior slope in order to increase the ROM of the knee. As Hernigou P, et al. reported an increase in aseptic loosening and ACL injury associated with increase posterior slope of the tibial [11].

When assessing radiographs, no patients in the group had evidence of component subsidence or pathological radiolucency. But the follow-up in this study is very early. Hopefully these results continue into the medium and long term. Ten components showed radiolucent lines which were defined as physiological radiolucency without clinical relevance. Tibrewal SB, et al. distinguished between the physiological radiolucency, which developed during the first year after surgery without any further progression and the pathological radiolucency caused by aseptic loosening or infection [29]. Gulati A, et al. also reported that 62 % of their cases showed physiological radiolucency [9]. There was no relationship being observed between BMI, age, gender, residual varus deformity or the status of the ACL and the incidence of radiolucency. In our study, 36 % (10 cases) of cases showed physiological radiolucency.

### Limitations

First, the number of cases in the study is small and the FU time is relatively short. Further study with large sample size cases with long term of FU should be done. Second, no comparative group was included in this study, which limits the evidence or our results. As this procedure is not very common and very technical demanding, after the promising results, we may design a study comparing this technique with other well-established techniques such as TKA or HTO.

## Conclusion

For the relatively young and active patients with primary ACL deficiency and secondary isolated femorotibial OA, and the patients with isolated femorotibial OA and acute ACL injury, the early and midterm clinical data have shown that combined surgery of Oxford UKA and ACL reconstruction has revealed promising results. However, long-term FU studies should be done in these patients.

## Abbreviations

ACL, anterior cruciate ligament; BMI, body mass index; FU, follow-up; HTO, high tibial osteotomy; KSS, knee society score; MRI, magnetic resonance imaging; OA, osteoarthritis; OKS, Oxford knee score; ROM, range of motion; TKA, total knee arthroplasty; UKA, unicompartmental knee arthroplasty.
